# De Novo Assembly, Annotation, and Characterization of Root Transcriptomes of Three Caladium Cultivars with a Focus on Necrotrophic Pathogen Resistance/Defense-Related Genes

**DOI:** 10.3390/ijms18040712

**Published:** 2017-03-27

**Authors:** Zhe Cao, Zhanao Deng

**Affiliations:** Gulf Coast Research and Education Center, Department of Environmental Horticulture, IFAS, University of Florida, 14625 County Road 672, Wimauma, FL 33598, USA; cjun01@gmail.com

**Keywords:** aroid, *Caladium*, root transcriptome, RNA-Seq, de novo assembly, disease resistance gene, defense-related gene

## Abstract

Roots are vital to plant survival and crop yield, yet few efforts have been made to characterize the expressed genes in the roots of non-model plants (root transcriptomes). This study was conducted to sequence, assemble, annotate, and characterize the root transcriptomes of three caladium cultivars (*Caladium* × *hortulanum*) using RNA-Seq. The caladium cultivars used in this study have different levels of resistance to *Pythium* *myriotylum*, the most damaging necrotrophic pathogen to caladium roots. Forty-six to 61 million clean reads were obtained for each caladium root transcriptome. De novo assembly of the reads resulted in approximately 130,000 unigenes. Based on bioinformatic analysis, 71,825 (52.3%) caladium unigenes were annotated for putative functions, 48,417 (67.4%) and 31,417 (72.7%) were assigned to Gene Ontology (GO) and Clusters of Orthologous Groups (COG), respectively, and 46,406 (64.6%) unigenes were assigned to 128 Kyoto Encyclopedia of Genes and Genomes (KEGG) pathways. A total of 4518 distinct unigenes were observed only in *Pythium*-resistant “Candidum” roots, of which 98 seemed to be involved in disease resistance and defense responses. In addition, 28,837 simple sequence repeat sites and 44,628 single nucleotide polymorphism sites were identified among the three caladium cultivars. These root transcriptome data will be valuable for further genetic improvement of caladium and related aroids.

## 1. Introduction

Caladium (*Caladium* × *hortulanum*) is an important ornamental aroid which is commonly used in containers, hanging baskets, and landscapes for their variably-shaped and colored leaves. The genus *Caladium* is native to the tropical regions of South and Central America, including Brazil, Colombia, Ecuador, and Peru. Modern cultivated caladiums are diploids with 2*n* = 2*x* = 30 chromosomes [1]. Recent molecular and cytological analyses have shown that cultivated caladiums are most likely to have originated from interspecific hybridizations between *Caladium bicolor* (Aiton) Vent. and *Caladium schomburgkii* Schott, which share the same chromosome number (30), similar nuclear DNA contents, and similar DNA fingerprints [1,2,3]. The great majority of commercial caladium plants are forced from tubers. More than 95% of the caladium tubers used in the world are produced in Florida [4].

A major issue affecting commercial caladium tuber and plant production is root rot caused by *Pythium* *myriotylum* Drech. Several other necrotrophic fungi including *Sclerotium rolfsii* Sacc. and *Rhizoctonia solani* Kuehn could also incite rot on caladium roots. *Pythium*-infected caladiums exhibit stunted growth, substantial losses of roots, and much-reduced tuber yield [4,5]. For example, Riding and Hartman [6] grew caladium tubers in *P. myriotylum*-infected substrates and observed that *Pythium* infection led up to 40% reduction in tuber yield. Caladium growers used to rely on methyl bromide fumigation for control of soil-borne pathogens [5], but methyl bromide has been phased out due to its severe side effect of depleting ozone in the atmosphere, and few alternative fumigants are available now. As a result, growers are facing much more severe losses from *Pythium* root rot.

Developing and using disease-resistant cultivars is an effective and environmentally-friendly approach to managing diseases in many crop production systems. For many soil-borne diseases caused by necrotrophic fungi/oomycetes that exhibit facultative pathogenicity and broad parasitic spectrums, the most common type of host resistance has been partial resistance [7]. Deng et al. [4,5] screened 42 caladium cultivars for *Pythium* root rot resistance and identified several commercial cultivars with moderate resistance, of which “Candidum” is particularly valuable, because it is also resistant to another necrotrophic pathogen, *Fusarium solani* (Mart.) Saa [8]. The continuous distribution of disease resistance level among the caladium cultivars evaluated in the reported studies indicates that the observed *Pythium* resistance is likely controlled by quantitative trait loci [4,5,9].

To date, our understanding of host resistance to necrotrophic pathogens has mainly come from studies in *Arabidopsis*, tomato, and rice [10,11,12]. These studies have shown that plant resistance toward necrotrophs is primarily mediated by recognition of pathogen-associated molecular patterns (PAMPs) via pattern recognition receptors (PRRs), signaling networks regulated by ethylene and jasmonic acid, and activation of biosynthesis of antimicrobial metabolites [13]. In *Arabidopsis*, dozens of genes encoding receptor-like kinases (RLKs) are essential for quantitative resistance to *Fursarium oxysporum* [14]. In rice, the gene *OsACS2*, which encodes 1-aminocyclopropane-1-carboxylic acid (ACC) synthase, plays an important role in conferring resistance to *Rhizoctonia solani* [15]. In tomato, expression of *Arabidopsis* thionin (an antimicrobial compound) gene *Thi2.1* significantly enhanced tomato resistance against Fusarium wilt [16]. Necrotrophic pathogens have caused severe losses to many crops in the world, and a better understanding of the type and expression of disease resistance and defense-related genes in plant roots could greatly facilitate further genetic improvement of crop resistance to necrotrophic pathogens.

Understanding the genetic basis for caladium resistance to necrotrophic pathogens, particularly *Pythium*, has been a very important objective in caladium genetic studies. Such knowledge would enable plant breeders to select the appropriate breeding parents and breeding crosses, and design effective and efficient selection schemes for the development of new caladium cultivars with enhanced resistance to necrotrophic pathogens. So far, little genomic or transcriptomic data are available for caladiums. The only publicly available DNA data in caladium are 99 simple sequence repeats (SSRs) reported by Gong and Deng [2], and 49 nucleotide sequences and 16 genes in the National Center for Biotechnology Information (NCBI) databases. None of the deposited DNA sequences were involved in disease resistance or defense. To discover putative disease resistance/defense genes in caladium, genomic and/or transcriptomic data are urgently needed.

In recent years, advances in next-generation sequencing (NGS) and assembly algorithms have resulted in a fast and deep transcriptome sequencing technique termed RNA-Seq. Recent development of de novo transcriptome assemblers has made RNA-Seq technology readily available for the study of non-model plants whose genomic sequences are largely unavailable [17]. Data from transcriptome sequencing has greatly facilitated genome-wide gene discovery, identification of genes involved in specific biosynthesis pathways, and development of molecular markers [18]. In recent years, de novo transcriptome sequencing has been reported in numerous non-model plant species for identifying putative genes involved in disease or stress responses. For example, Zhang et al. [19] identified 250 nucleotide-binding site and leucine-rich repeat (NB-LRR) transcripts from shoots, leaves and flower buds in two *Camellia* species. In *Oryza officinalis*, He et al. [20] discovered disease resistance genes involved in signaling and plant hormone regulation pathways using leaves, stems and roots. In gerbera, Fu et al. [21] sequenced leaf transcriptomes and found 137 transcripts that were related to the phenylpropanoid biosynthesis pathway that impacts disease resistance. Additionally, transcriptome sequencing data have been used for the development of SSR and single nucleotide polymorphism (SNP) molecular markers in many plant species [22,23].

Herein, the root transcriptomes of three caladium cultivars (“Candidum”, “Gingerland”, and “Miss Muffet”) were sequenced using the paired-end sequencing protocol and the Illumina HiSeq 2000 platform and were assembled de novo. The three cultivars were selected because they are very important breeding parents, and most importantly, they displayed significant differences in *Pythium* root rot resistance. “Candidum” is moderately resistant to *Pythium* root rot, while “Gingerland” and “Miss Muffet” are highly susceptible to this disease [4,5,9]. The objectives of this study were to (1) assemble, annotate and characterize the root transcriptomes of these caladium cultivars; (2) identify transcripts that are likely involved in disease resistance/defense pathways; and (3) identify SSR and SNP sites in caladium transcriptomes. This is the first transcriptome study in caladium; the data generated here will be of significant value for development and use of molecular markers in genetic improvement of caladium.

## 2. Results and Discussion

### 2.1. Quality Control and De Novo Transcriptome Assembly

A total of 68,000,078, 50,980,474, and 67,583,936 raw reads were obtained for “Candidum”, “Gingerland”, and “Miss Muffet”, respectively (Table 1). The FastQC v0.11.4 analysis of raw sequences showed that the percentage of Q20 (sequencing quality score 20, representing a call accuracy of 99%) reads ranged from 96.84% (“Candidum”) to 96.97% (“Gingerland”) and the percentage of GC contents in these reads varied from 49.20% (“Candidum”) to 49.84% (“Miss Muffet”). After filtering out of adaptors and low-quality reads, 61,439,682 (“Candidum”), 46,148,492 (“Gingerland”), and 61,034,284 (“Miss Muffet”) clean reads were retained. The high Q20 value (>95%) indicated that the sequencing data of the three caladium libraries were of high quality and suitable for conducting assembly.

De novo transcriptome assembly of the clean reads in Trinity yielded 255,215 (“Candidum”), 232,333 (“Gingerland”), and 258,116 (“Miss Muffet”) contigs, respectively (Table 2). The average length and N50 length of these contigs were similar among “Candidum”, “Gingerland”, and “Miss Muffet”: average lengths from 292 bp (“Miss Muffet”) to 299 bp (“Gingerland”), and N50 lengths from 463 bp (“Miss Muffet”) to 492 bp (“Gingerland”). Of these contigs, 66.81% (“Gingerland”) to 67.56% (“Candidum”) were <200 bp, 21.20% (“Candidum”) to 21.45% (“Gingerland”) were from 200 to 500 bp, and 11.19% (“Miss Muffet“) to 11.24% (“Candidum”) were >500 bp (Table S1).

Unigene assemblies of the three caladium cultivars were constructed separately, resulting in a total of 133,737 (46,962 clusters and 86,775 singletons), 122,994 (43,721 clusters and 79,273 singletons), and 135,589 (47,844 clusters and 87,745 singletons) unigenes for “Candidum”, “Gingerland”, and “Miss Muffet”, respectively, with an average length of 729 bp (“Candidum”), 736 (“Gingerland”) and 717 (“Miss Muffet”) bp, and an N50 length of 1525 bp (“Candidum”), 1514 (“Gingerland”), and 1487 bp (“Miss Muffet”) (Table 2).

Merging the unigenes from the three caladium cultivars resulted in a total of 137,354 (62,353 clusters and 75,001 singletons) unigenes for caladium, with an average length of 999 bp and an N50 length of 1755 bp (Table 2). The length distribution of these unigenes was as follows: ≈62.0% unigenes were 100–500 bp, ≈14.0% unigenes were 500–1000 bp, ≈14.5% unigenes were 1000–2000 bp, and ≈8.9% unigenes were >2000 bp (Table S2).

So far, there has been no report of transcriptome assembly in caladium. In *Amorphophallus*, another aroid species, Zheng et al. [24] sequenced and generated 135,822 unigenes with an average length of 523 bp and an N50 length of 635 bp. Overall, this set of assembled caladium unigenes were longer than those of *Amorphophallus*.

### 2.2. Functional Annotation of Assembled Unigenes and Coding Sequence (CDS) Prediction

To annotate assembled unigenes, the NCBI BLAST+ 2.2.28 was used to conduct sequence similarity search (*E*-value cutoff of ≤10^−5^) in public databases including the NCBI non-redundant protein (NR) and nucleotide sequence (NT) databases, the Swiss-Prot, the Kyoto Encyclopedia of Genes and Genomes (KEGG), Clusters of Orthologous Groups (COG), or Gene Ontology (GO) database (Table 3). Out of the 137,354 assembled sequences, 68,827 (50.1%), 52,233 (38.0%), and 47,907 (34.9%) were successfully annotated in NR, NT and Swiss-Port databases, respectively. Further, 46,406 (33.8%), 31,417 (22.9%), and 48,417 (35.2%) unigenes were then annotated in KEGG, COG, and GO databases, separately. A total of 71,825 unigenes (52.3%) were annotated in at least one of these six databases (Table 3).

**Figure 1:**
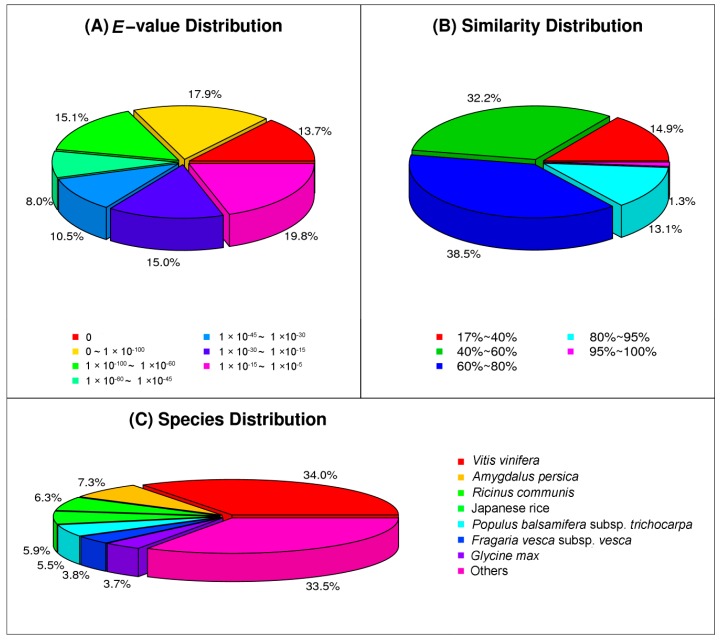
Characterization of caladium unigenes via similarity searches against the NR database. (**A**) *E*-value distribution of BLAST hits for the assembled unigenes; (**B**) similarity distribution of BLAST hits for the assembled unigenes; and (**C**) species distribution of the top BLAST hits for the assembled unigenes.

Out of the 68,827 unigenes annotated in NR, 43,016 (65.2%) unigenes had homology with the deposited reference sequences at *E*-value < 1.0 × 10^−30^ (Figure 1A), 9911 (14.4%) unigenes showed 80% or greater sequence similarity to reference genes, and 26,498 (38.5%) unigenes had 60% to 80% sequence similarity to the reference genes (Figure 1B). The unigenes assembled from caladium root transcriptomes were most homologous to genes of *Vitis vinifera* (23,401, 34.0%), remotely followed by *Amygdalus persia* (5024, 7.3%), *Ricinus communis* (4336, 6.3%), Japanese rice (4060, 5.9%), *Populus balsamifera* (3785, 5.5%), *Fragaria vesca* (2615, 3.8%), and *Glycine max* (2546, 3.7%) (Figure 1C). The relatively high levels of similarity between caladium unigenes and the genes of distantly related *V. vinifera* were surprising as caladium unigenes were expected to be more homologous to genes of rice or other monocotyledonous plants. Nevertheless, a similar phenomenon was observed in other aroid transcriptome studies. For example, 32.7% of *Anthurium* unigenes shared highest homologies to *V. vinifera* genes [25]. The exact causes of this phenomenon remains to be elucidated.

#### 2.2.1. Gene Ontology (GO) Classification

**Figure 2:**
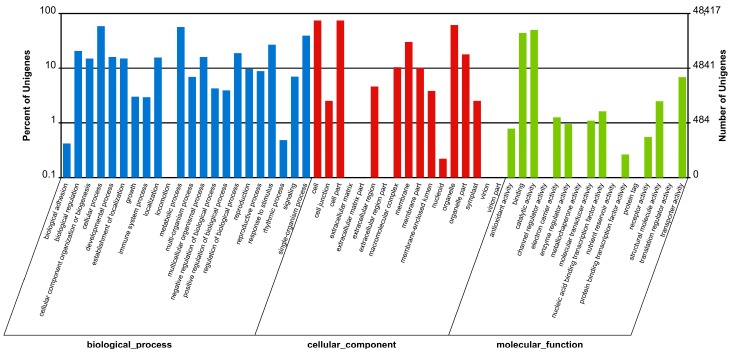
Gene ontology (GO) classification of annotated caladium unigenes. Results were grouped into three main categories: Biological Process, Cellular Component, and Molecular Function. The right *y*-axis showed the number of unigenes corresponding to each subcategory, and the left *y*-axis showed the percentage of unigenes involved in each specific subcategory.

To functionally classify the assembled unigenes, sequences that were annotated in the NR database were then subjected to Gene Ontology (GO) analysis. Results showed that 68,827 caladium unigenes fell into three main categories, including Biological Process, Cellular Component, and Molecular Function (Figure 2 and Table S3). In the Biological Process category, the Cellular Process (28,428 unigenes, 16.96%), Metabolic Process (27,380 unigenes, 16.33%), and Single-Organism Process (18,997 unigenes, 11.33%) were the three primary subcategories. With respect to the Cellular Component category, Cell (35,952 unigenes, 25.38%), Cell Part (35,948 unigenes, 25.37%), and Organelle (29,813 unigenes, 21.04%) were the most enriched subcategories, and in the Molecular Function category, the top three subcategories were Catalytic Activity (24,305 unigenes, 45.39%), Binding (21,486 unigenes, 40.13%), and Transporter Activity (3322 unigenes, 6.20%).

**Figure 3:**
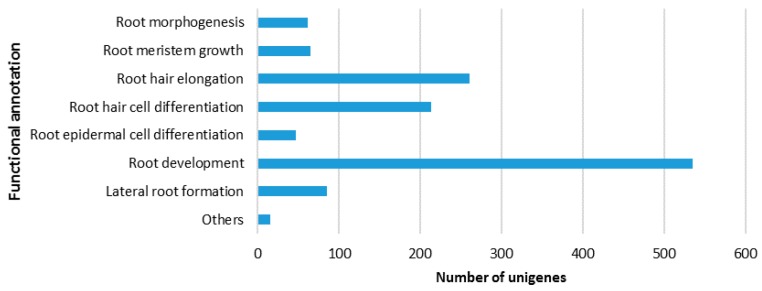
Root-specific unigenes identified in the three caladium root transcriptomes. The *y*-axis on the right indicates the functional annotation categories, and the *x*-axis on the bottom shows the number of unigenes falling into each functional annotation category.

Out of the 68,827 unigenes annotated for GO classifications, 1284 unigenes seem to be root-specific (Table S4). A great majority of them (1284, 98.75%) could be grouped into seven functional categories, including root morphogenesis (62 unigenes, 4.83%), root meristem growth (65 unigenes, 5.07%), root hair elongation (261 unigenes, 20.33%), root epidermal cell differentiation (47 unigenes, 3.67%), root development (535 unigenes, 41.67%), and lateral root formation (85 unigenes, 6.62%) (Figure 3). A small number of the root-specific unigenes (16, 1.25%) could not be assigned to specific functional categories, thus grouped into “Others”. A total of 103 root-specific unigenes are related to disease resistance or plant defense (Table S5).

#### 2.2.2. Clusters of Orthologous Groups (COG) Classification

**Figure 4:**
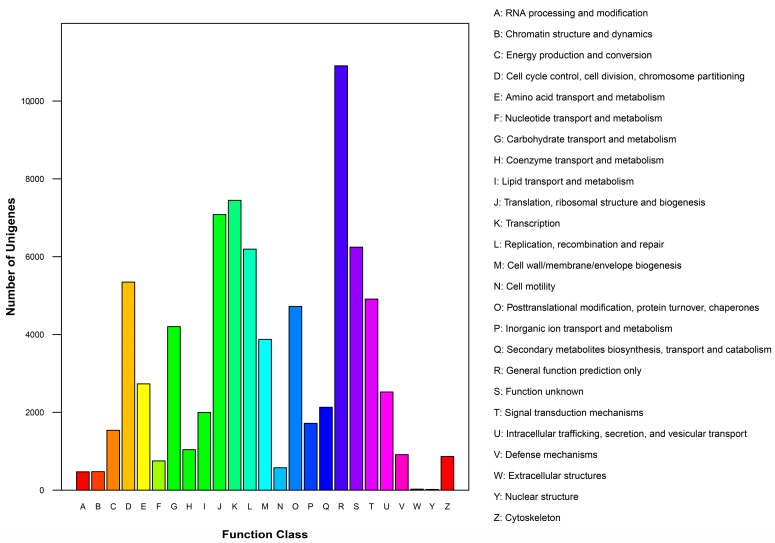
Clusters of Orthologous Groups (COG) classification of assembled caladium unigenes. The COG function classification of these unigenes fell into 26 categories (*x*-axis). The *y*-axis showed the number of caladium unigenes corresponding to each category.

To predict the putative function of unigenes, all caladium unigenes were compared with the Clusters of Orthologous Groups (COG) database. As shown in Figure 4, the largest number of unigenes had “general function prediction only” (10,905, 13.85%), followed by “transcription” (7448, 9.56%), “translation, ribosomal structure, and biogenesis” (7082, 9.46%), “function unknown” (6245, 7.93%), “replication, recombination, and repair” (6194, 7.87%), “cell cycle control, cell division, chromosome partitioning” (5347, 6.79%), “signal transduction mechanisms” (4910, 6.24%), and other 20 functions, from “posttranslational modification, protein turnover, chaperones” (4722, 5.99%) to “nuclear structure” (19, 0.02%) (Table S6).

#### 2.2.3. Kyoto Encyclopedia of Genes and Genomes (KEGG) Classification

To identify the active biological pathways in caladium roots, the assembled unigenes were analyzed using the Kyoto Encyclopedia of Genes and Genomes (KEGG) Pathway database. Results showed that 46,406 (64.6%) unigenes could be assigned to 128 KEGG metabolic pathways. The top ten pathways are listed in Table 4. Among the primary KEGG categories, “metabolism pathways” had the highest percentage of caladium unigenes (26.9%), followed by “RNA transport” (11.6%), “biosynthesis of secondary metabolites” (10.3%), “mRNA surveillance pathway” (9.0%), “glycerophospholipid metabolism” (7.8%), and “endocytosis” (7.7%).

#### 2.2.4. CDS Prediction

**Figure 5:**
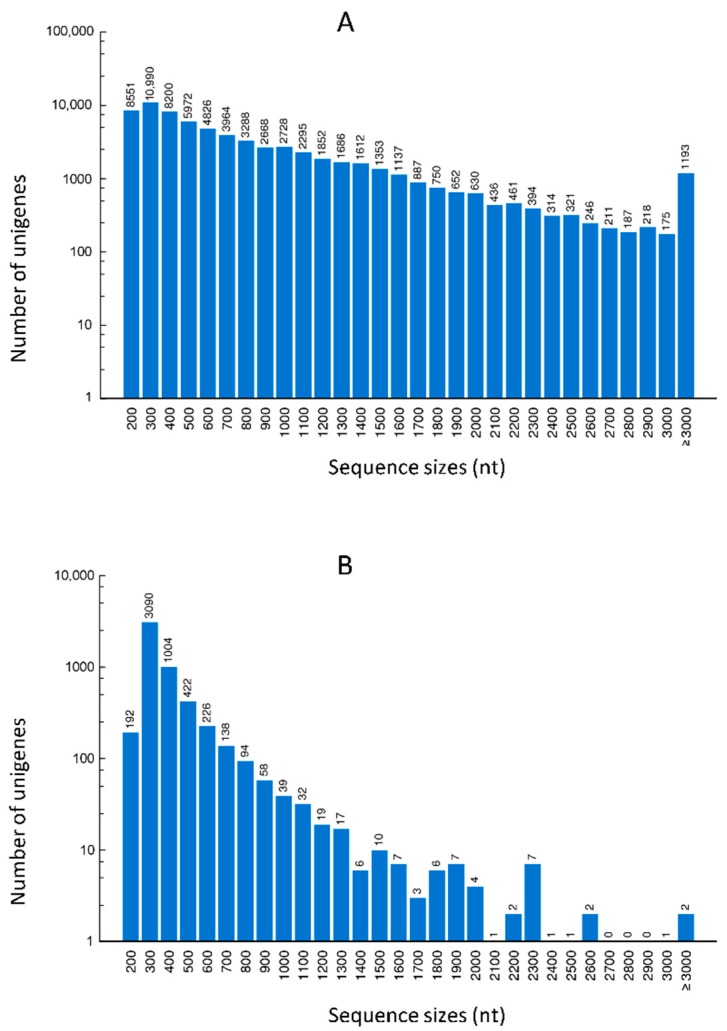
Length distribution of CDS predicted in the NR, Swiss-prot, KEGG and COG databases (**A**), or by the ESTscan (**B**).

The protein coding sequence (CDS) and the amino acid sequence of all assembled unigenes were predicted using NCBI BLAST 2.2.28+ and ESTScan (version 3.0.2). Unigenes were firstly aligned in the NR, Swiss-prot, KEGG, and COG databases for CDS prediction. The non-hit unigenes were then subjected to ESTScan (version 3.0.2). In total, the CDS of 68,197 unigenes were successfully predicted in the databases described above. The majority of predicted CDS were in the ranges of 200–500 bp (49.4%), followed by CDS of 600–1000 bp (25.6%), CDS of 1100–2000 bp (18.8%), and CDS of >2000 bp (6.0%) (Figure 5A). The majority of CDS of unigenes (99.2%) predicted by the ESTScan software were less than 500 bp (Figure 5B).

#### 2.2.5. Comparison with Reported Transcriptome Assemblies

The caladium root transcriptome as assembled above consists of 137,354 transcripts and 71,825 unigenes. This number of unigenes largely exceeds the number of gene models in the majority of sequenced plant genomes (20,000 to 40,000) [26]. This phenomenon seems to be not uncommon in transcriptome assemblies and analyses of other aroids [25] and other non-model plants [26]. In *Anthurium*, a high proportion of short unigenes (69.8%) and a large number of unigenes (111,268) were reported [25]. Several factors might have contributed to this phenomenon. The lack of reference genome sequences in non-model plants makes it difficult to reconstruct full-length gene transcripts from short sequence reads and read alignments. Consequently, multiple unigenes representing the same genes might have failed to be assembled and were treated as independent unigenes, resulting in large numbers of short unigenes and excessive unigene numbers [26]. Alternative splicing of protein-coding genes and expression of transposable elements and pseudogenes will certainly result in larger numbers of transcripts than the number of gene models annotated based on genome sequences. A third potential cause of the large number of unigenes in the current caladium root transcriptome assembly might be the relative large size of the caladium genome (≈4.5 Gb) [1]. When a species possesses a large sized and highly duplicated genome, the number of unigenes tended to be larger [26]. In *Amorphophallus bulbifer*, an aroid with a genome of ≈9.0 Gb, 119,678 unigenes was reported for its leaf transcriptome. In *Allium cepa*, a plant with a nuclear genome of 16 Gb, its bulb transcriptome consisted of 115,251 unigenes [27].

Nearly 50% of the caladium unigenes were short (200–500 bp). This was a major and frequent issue in de novo assembly of transcriptomes of non-model plants [28,29,30]. Several factors might have contributed to the presence of large numbers of short unigenes, including under-estimated sequencing depth needs, the use of stringent parameters in the assembly process, lack of reference genomes for read mapping, and highly complex transcriptomes. Deep sequencing using the single molecule real-time sequencing (SMRT) technology may be a very effective approach to extending unigenes to full-length transcripts.

De novo assembly of a soybean root transcriptome resulted in 37,287 contigs and 35,081 unigenes [31]. In *Callerya speciosa*, a total of 161,926 contigs and 111,706 unigenes were reported for its de novo assembled root transcriptome [32]. A de novo root transcriptome of Louisiana iris consisted of 525,498 transcripts and 313,958 unigenes [33]. These studies, as well as ours, on caladium suggest that the number of assembled contigs and the number of annotated unigenes could vary remarkably among root transcriptomes of different plant species. This variation may indicate a high level of diversity among plant species in root transcriptome (and metabolic) activities.

### 2.3. Identification of Putative Unigenes Involved in Resistance and Defense against Necrotrophic Pathogens

**Figure 6:**
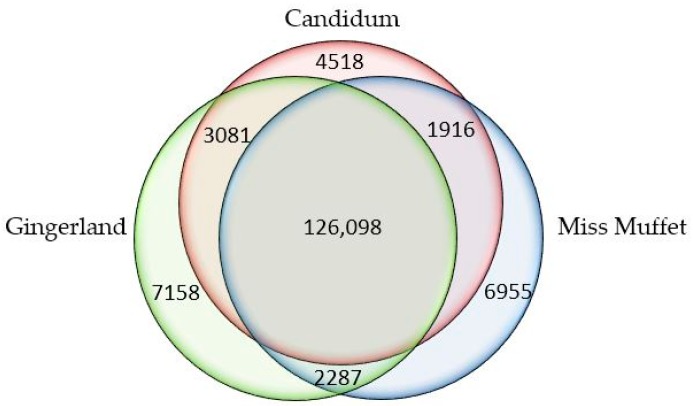
Venn diagram showing the number of specific and common unigenes in three caladium cultivars, “Candidum”, “Gingerland”, and “Miss Muffet”.

In response to attacks by necrotrophic pathogens, the innate immune system of plants perceives PAMPs with PRRs and activates PAMP-triggered immunity (PTI) against the pathogens [13]. PTI involves a number of cellular processes, including accumulation of reactive oxygen species and nitric oxide, modulation of hormone level, secretion of antimicrobial compounds, and reinforcing of the cell wall [34]. In this study, we compared unigenes in the root transcriptome of “Candidum” (moderately resistant to *P. myriotylum*) with those in the root transcriptomes of “Gingerland” and “Miss Muffet” (both highly susceptible to the pathogen) and identified 4518 unigenes that were present only in the root transcriptome of “Candidum” (Figure 6 and Table S7). These unique unigenes were examined for potential involvement in disease resistance and plant defense, in particular, (1) early signal perception and transduction in plant-pathogen interactions; (2) hormone regulation during plant defense responses to necrotrophic pathogens; and (3) secondary metabolites with antimicrobial activities in plant defense (Table 5).

#### 2.3.1. Early Signal Perception and Transduction in Plant-Pathogen Interactions

Recognition of PAMPs through membrane-localized PRRs during plant-pathogen interactions can induce an immune response syndrome [35]. Previous studies have indicated that a series of plant RLKs could be PRRs for plant immune responses [36]. Lack of RLKs could attenuate plant responses to pathogens, resulting in disease susceptibility [37]. All RLKs are transmembrane proteins, each containing a signal sequence, a putative extracellular domain at the amino-terminal, and an intracellular kinase domain at carboxyl-terminal [38]. Previous studies have shown that RLKs participate in many biologically important processes that confer disease resistance [39].

Of the 4518 unigenes found only in “Candidum”, 50 unigenes can be translated into RLKs, including one l-type lection receptor kinase (LecRK), one cysteine-rich receptor-like kinase (CRK2), 12 receptor-like serine/threonine-protein kinases (STKs), 31 proline-rich receptor-like protein kinases (PERKs), and five possible RLKs (Table 5 and Table S8). LecRKs are important immune receptors that bind to the effectors produced by *Phytophthora infestans* [40]. Previous studies have shown that *LecRK* genes confer resistance in *Arabidopsis* to several necrotrophic oomycetes including *Phytophthora brassicae* and *Botrytis cinerea* [41,42]. The one *LecRK* homolog in “Candidum” may play a positive role in its disease resistance. CRKs are one of the largest classes of RLKs in plants [43]. Studies in *Arabidopsis* have shown that CRKs regulate plant development, stress adaptation, and disease resistance [44]. Yang et al. [45] found that a novel *CRK* homolog (*TaCRK1*) is involved in wheat resistance to *Rhizocotonia cerealis* and the expression levels of *TaCRK1* were significantly higher in moderately resistant lines than in highly susceptible lines. In “Candidum” root transcriptome, only one *CRK* homolog (*CRK2*) was observed (Table 5). Another important type of plant receptor-like kinases are STKs, which can phosphorylate the hydroxyl group (OH) of serine or threonine residues, resulting in functional alterations of target proteins [46]. The phosphorylated proteins play an important role in disease resistance pathway by triggering a series of downstream activities including protein modulation, protein-protein interaction, transcription factor activation, and secretion of antimicrobial metabolites [47]. It has been shown that two wheat *STK* homologs, *Tsn1* and *Stpk-V*, are the key genes conferring resistance against biotrophic *Blumeria graminis* (wheat powdery mildew) and the necrotrophic pathogens *Stagonospora nodorum* (Stagonospora nodorum blotch) and *Pyrenophora tritici-repentis* (tan spot) [46,48]. In this study, 12 *STK* homologs were observed exclusively in the “Candidum” root transcriptome (Table 5). In addition, we found that a majority of RLKs in the “Candidum” (60%) root transcriptome belong to PERKs. In *Arabidopsis*, PERKs represent a small family of RLKs, all sharing an extracellular domain, a transmembrane domain, and a kinase domain [38]. PERKs in *Arabidopsis* roots are involved in callose and cellulose deposition; PERK1 mutants have lost their ability to accumulate callose and cellulose in cell walls [49]. Silva and Goring [50] found that PERK1 could response to both mechanical stresses and infection by fungal pathogens and may have a function in the general perception of wounds or recognition of pathogen stimuli. In the “Candidum” root transcriptome, 31 *PERK* homologs were observed (Table 5), and these *PERKs* may be good candidates for further gene expression studies. There were five possible *RLK*-like unigenes (Table 5) that could not be further annotated, suggesting that these *RLK*-like unigenes may represent unique transcripts in caladium and their functions remain to be studied.

After plant perception of PAMPs, signals from the PRR complexes are transferred to downstream targets through several secondary messengers, including influx of calcium (Ca^2+^), reactive oxygen species (ROS) burst, and activation of mitogen-activated protein kinases (MAPKs) [51]. The calcium influx is an early step in the signal transduction processes [52]. Ca^2+^ influx can be sensed by several stimulus-specific Ca^2+^-binding sensors such as calcium-dependent protein kinases (CPKs) and Calmodulins (CaMs). Previous studies have shown that transgenic potato plants containing *StCDPK4* and *StCDPK5* (*CPK* homologs) gain resistance to *Phytophthora infestans* [53]. A wheat *CPK* homolog (*TaCPK7*-D) is involved in determining resistance to necrotrophic pathogen *Rhizoctonia cerealis*. Here, we identified eight CPKs homologs only in “Candidum“ (Table 5 and Table S9) and suspect that they may be important signal sensors which modulate resistance to necrotrophic pathogens. CaMs and CaM-like (CML) genes could trigger plant defense responses to a broad range of pathogens. In tobacco, a *CML* gene, *NtCaM13*, is a key gene for resistance to the necrotrophic pathogens *Pythium aphanidermatum* and *Rhizoctonia solani* [54]. Four *CaM* gene homologs were identified among “Candidum” unigenes (Table 5). ROS are important signals to modulate the expression of defense-related genes to fungal pathogen attacks [55]. The production of ROS involves a number of oxidative enzymes such as the NADPH (nicotinamide adenine dinucleotide phosphate) oxidase, the superoxide dismutase, the cell wall peroxidase, the oxalate oxidase, the lipoxygenase, and the polyamine oxidase [56]. These enzymes work collaboratively or separately to initiate a series of responses including cell wall remodeling, signal transduction, programming cell death, and post-translation regulation to resist pathogens [56]. So far, our understanding of the roles ROS play in plant roots and resistance against necrotrophic pathogens is still rudimentary [56]. In banana, studies have shown that a NADPH oxidase homolog (*Rboh*) confers resistance against the necrotrophic pathogen *Fusarium oxysporum*. In *Arabidopsis* and cotton, *F. oxysporum*-resistant lines accumulated ROS faster than the susceptible lines [57,58]. In this study, we identified five putative ROS-related unigenes (superoxide dismutase) that were exclusively expressed in “Candidum” (Table 5). MAPK activation is another early signaling activity after plants sense the attack from pathogens [59]. Plant MAPK cascades transfer signals from PRRs to downstream components [60]. In *Arabidopsis*, MAPK cascades involve several MAPKs such as MEKK1, MKK1/MKK2 (two redundant MAPKKs), MPK4/MPK5 (two redundant MAPKs), and MPK3/MPK6 (two partially redundant MAPKs). Previous studies have shown that MPK3, MPK4, MPK6, MKK1, MKK2, and MEKK1 are involved in innate immune responses against necrotrophic pathogens [61]. Of 4956 unigenes only observed in the “Candidum” root transcriptome, there were eight *MAPK* homologs including three *MPK1*, two *MPK2*, one *MPK4*, one *MPK5*, and one *MPK14* homolog (Table 5).

#### 2.3.2. Hormone Regulation during Plant-Pathogen Interactions

Increased biosynthesis of hormones is an important plant defense response to invading pathogens [37]. In plants, ethylene (ET) and jasmonic acid (JA) are two important hormones involved in resistance against necrotrophic pathogens. *S*-adenosylmethionine (*S*-AdoMet) and 1-aminocyclopropane-1-carboxylic acid (ACC) are two precursors of ethylene [62]. Enzymes catalyzing ET biosynthesis reactions include ACC synthase (ACS) and ACC oxidase (ACO) [62]. Among the annotated unigenes in “Candidum”, both key enzymes participating in ET synthesis were identified (Table 5 and Table S10). Synthesized ethylene can be then perceived and transduced by ethylene receptors to trigger downstream biological responses. There are several types of ethylene receptors in *Arabidopsis*, such as ethylene receptors (ETRs), ethylene response sensors (ERSs), and ethylene insensitive (EINs) [63]. However, the roles ethylene receptors play in necrotrophic pathogen resistance seem to be different depending on plant species and the types of pathogens. In *Arabidopsis*, the *ein2* (ethylene insensitive) mutant lines displayed increased susceptibility to the necrotrophic pathogen *Botrytis cinerea* [64], while in soybean, the *etr1* and *etr2* (ethylene-resistant) mutant lines showed increased resistance against *Phytophthora sojae* [65]. In this study, we observed 14 *ETR* homologs that were expressed in the three caladium cultivars. One *ETR*-like unigene was expressed exclusively in the “Candidum” root transcriptome; this unigene could be a good candidate for future studies.

JA is another important hormone in plant defense against many necrotrophic pathogens [66]. It has been reported that exogenous applications of methyl jasmonate to *Arabidopsis* enhance plant resistance to root rot disease caused by the necrotrophic pathogen *Pythium mastophorum* [67]. Cohen et al. [68] found that methyl jasmonate-treated potatoes showed increased resistance to *Phytophthora infestans*. Studies in tomato have shown that the *CORONATINE-INSENSITIVE1* gene (*COI1*), the acyl-CoA oxidase gene (*ACX1A*), the *suppressor of prosystemin-mediated responses2* (*SPR2*), and the *defenseless1* gene (*def1*) are involved in JA-induced defense responses against necrotrophic pathogens such as *Botrytis cinerea* [37]. Tomato plants with either one of these genes mutated could not correctly process JA synthesis, and JA signal perception or transduction, resulting in disrupted immune responses and susceptible plants [37]. We found that 55 unigenes were potentially involved in JA synthesis and expressed in all three caladium cultivars. One *ACX1A* homolog was expressed only in “Candidum” (Table 5), which suggests that this unigene may be a good candidate for further gene expression studies.

#### 2.3.3. Secondary Metabolites with Antimicrobial Activities

Plants can secrete secondary metabolites around their rhizosphere to inhibit or repel soil-borne pathogens. In general, phenolics and terpenoids are two primary classes of root-secreted antimicrobial metabolites [69]. Common root-secreted phenolic compounds include phenylpropanoids and flavonoids [70]. Many phenylpropanoids from a broad range of plant species have shown antimicrobial activities [71]. Phenylpropanoids in chickpea roots affect resistance against the necrotrophic pathogen *Fusarium oxysporum*; resistant chickpea varieties produce significantly more phenylpropanoids than susceptible ones [72]. Lanoue et al. [71] found that phenylpropanoids were constitutively secreted by the barley root system and inhibited *Fusarium graminearum* spore germination. In “Candidum“, we found two unigenes that are likely involved in phenylpropanoid synthesis. Flavonoids are often one of the largest groups of phenolics in root exudates [69]. Studies in maize have indicated that high levels of flavonoids are associated with an increased defensive state in its roots [73]. We identified four unigenes in “Candidum” that are likely involved in flavonoid synthesis (Table 5 and Table S11).

Terpenoids are another large group of compounds in root exudates that have antimicrobial activities [74]. Some volatile terpenoids emitted from roots could act as a direct barrier against pathogens [69]. In *Arabidopsis*, Steeghs et al. [75] found a below-ground volatile compound (monoterpene 1,8-cineole) which had direct effects on *Pseudomonas syringae*. Kapulnik et al. [76] observed that strigolactones released from roots inhibited the growth of several soil-borne pathogens, including *Fusarium oxysporum*, *Fusarium solani*, and *Macrophomina phaseolina*. We identified four unigenes in the “Candidum” root transcriptome that are likely involved in the terpenoid synthesis pathway. Derivatives of tryptophan are another important antimicrobial root exudates. Typical examples of tryptophan derivatives include camalexin, which is produced by *Arabidopsis* and exhibits antimicrobial activities to a wide range of soil-borne pathogens including *Pythium sylvaticum*, *Rhizoctonia solani*, and *Sclerotinia sclerotiorum* [77,78]. In the “Candidum” root transcriptome, we found nine tryptophan synthesis-related unigenes (Table 5 and Table S11), which may be involved in resistance against soil-borne necrotrophic pathogens. One of these unigenes is root-specific.

### 2.4. Simple Sequence Repeat (SSR) and Single Nucleotide Polymorphism (SNP) Discovery

Transcriptome sequencing data have become a very important resource for discovering nucleotide sequence polymorphisms and developing molecular makers. SSRs and SNPs are two very important types of nucleotide sequence polymorphisms, and each has its own characteristics: SSRs are multi-allelic and hyper-variable, while SNPs are the most abundant type [79]. Molecular markers based on SSRs and SNPs have been widely used in plants for a wide range of research and practical purposes, including estimation of genetic diversity, determination of phylogenetic relationship, development of genetic linkage maps, identification of quantitative trait loci (QTL), association studies, marker-assisted breeding, and cultivar identification and protection [80]. In many uses, SSR and SNP-based molecular markers can complement each other.

To reveal SSRs in caladium transcriptomes, all assembled unigenes were subjected to the MISA software for screening putative SSR motifs. As a result, 28,837 SSR repeats were discovered, including 2594 mono-, 15,504 di-, 9583 tri-, 443 quad-, 346 penta-, and 367 hexa-nucleotides (Table 6). The most abundant type of SSR motifs was AG/CT (43.2%), followed by A/T (7.8%), AGG/CTT (7.5%), and AC/GT (6.1%) (Table 6). The 8988 unigenes that contain these SSRs were subjected to the Primer 3 software for primer designing, which resulted in 44,922 pairs of primers for use in caladium (Table S12).

To detect SNPs, the unigenes of “Candidum”, “Gingerland”, and “Miss Muffet” were merged to generate a set of reference sequences. SNP sites were called for “Candidum”, “Gingerland”, and “Miss Muffet” by mapping their clean reads to the set of reference sequences. As a result, 281,728, 244,685, and 270,217 SNP sites were identified for “Candidum”, “Gingerland”, and “Miss Muffet”, respectively (Table S13). The frequency of SNPs in these unigenes ranged from 1 per 487 bp in “Candidum” to 1 per 560 bp in “Gingerland”. The most abundant SNPs were of the transition-type (about 60.1%), cytosine to thymine (C ↔ T) or adenine to guanine (A ↔ G), followed by the transversion-type (A ↔ C, A ↔ T, C ↔ G, or G ↔ T) (39.9%) (Table 7 and Table S13).

Molecular markers have become a highly valuable tool in genetic improvement of plant resistance to various pathogens [81]. The main bottleneck that constrains breeding efforts to improve *Pythium* root rot resistance in caladium has been the low efficiency in phenotyping large breeding populations and the difficulty in obtaining reliable data for resistance to *Pythium* root rot. The availability of molecular DNA markers closely linked to or associated with caladium root rot resistance will empower breeders to implement highly desired marker-assisted selection in caladium. The putative disease resistance/defense genes and the large numbers of SNPs and SSRs identified in caladium root transcriptomes will serve as a very important genomic resource for developing molecular markers, linkage map construction, association studies, identification and validation of caladium QTL linked to *Pythium* root rot resistance. Such a marker-assisted selection strategy has been successfully implemented in many agronomic and horticultural plant species and has resulted in enhanced resistance to a wide spectrum of pathogens [82]. It is expected that application of this strategy will speed up the development and deployment of new *Pythium*-resistant caladium cultivars.

## 3. Material and Methods

### 3.1. Plant Material

Tubers (approximately 6 to 9 cm in diameter) of caladium cultivars “Candidum”, “Gingerland”, and “Miss Muffet” were planted individually in plastic containers (15 cm diameter) filled with coarse vermiculite (Palmetto Vermiculite Company, Woodruff, SC, USA) and irrigated with an overhead misting system (once a day). Plants sprouted from the tubers were grown in a greenhouse at the University of Florida’s Gulf Coast Research and Education Center, Wimauma, FL, USA. Temperatures inside the greenhouse were maintained between 21 °C (night) and 30 °C (day). Five grams of a controlled-release fertilizer (Osmocote^®^; The Scotts Miracle-Gro Company, Marysville, OH, USA) was applied to the top of the potting mix in each container to provide nutrients for the plants. Two months after the tubers were planted, roots were collected from four plants per cultivar, combined into a 50-mL centrifuge tube, and frozen with liquid nitrogen. The frozen root tissues were stored at –86 °C in a deep freezer (NuAire Corporate, Plymouth, MN, USA) until use.

### 3.2. RNA Extraction and Quality Control

Total RNA was isolated from the frozen root tissues using the pBiozol Total RNA Extraction Reagent (BioFlux, Hangzhou, China) following the manufacturer’s instructions. RNA concentration, RNA integrity number (RIN), and the 28S/18S ratio were determined on an Agilent 2100 Bioanalyzer (Agilent Technologies, Santa Clara, CA, USA) at the BGI (Beijing Genomics Institute, Shenzhen, China).

### 3.3. Illumina cDNA Library Preparation and Transcriptome Sequencing

Three cDNA libraries were prepared at BGI using the TruSeq^TM^ RNA Sample Prep Kit v2 (Illumina, Inc., San Diego, CA, USA). Approximately 200 ng of total RNA for each sample were purified with oligo-dT (25) beads. The purified poly(A)-containing mRNAs were fragmented into small pieces of 120–200 bp using the Elute, Prime, and Fragment Mix reagents from Illumina TruSeq™ RNA Sample Prep Kit v2 at 94 °C for 8 min. First-strand cDNA was synthesized using the First Strand Master Mix and SuperScript II from Invitrogen (Carlsbad, CA, USA) and the following PCR program: 25 °C for 10 min, 42 °C for 50 min, and 70 °C for 15 min. Second-strand cDNA was synthesized using the Second Strand Master Mix from Invitrogen at 16 °C for 1 h. End repair was performed using the End Repair Mix from Invitrogen at 30 °C for 30 min and the Ampure XP beads (Beckman Coulter, Brea, CA, USA). The resulting cDNA fragments were adenylated using the A-Tailing Mix from Illumina at 37 °C for 30 min. Subsequently, the cDNA fragments were ligated to the RNA Index Adapter at 30 °C for 10 min using the Ligation Mix from Illumina and then purified with the Ampure XP beads (Beckman Coulter). Purified cDNA fragments were enriched with 10 cycles of PCR: 98 °C for 10 s, 60 °C for 30 s, and 72 °C for 30 s. The resulting PCR products were purified with Ampure XP beads (Beckman Coulter). The final libraries were quantitated using an Agilent 2100 Bioanalyzer (Agilent Technologies). Prepared cDNA libraries were amplified on a cBot^TM^ system (Illumina) to generate clusters on the flow cell using the TruSeq PE Cluster Kit V4-cBot-HS (Illumina) and then subjected to the 125 bp paired-end sequencing on a HiSeq 2000 system (TruSeq SBS KIT-HS V4, Illumina) at BGI.

### 3.4. Raw Sequencing Data Trimming and De Novo Transcriptome Assembly

Raw sequence reads were cleaned using the filter_fq software (an internal version of BGI) to remove adaptors, reads with more than 5% unknown (N) nucleotides, and low quality reads which had more than 20% of the nucleotides scored ≤10. De novo assembly of clean reads was performed using the short reads assembling software, Trinity (version 20130225) [83], and its default parameters, with the following changes: min contig length = 100, reinforcement distance = 85, min glue = 3, group pair distance = 250, and min kmer cov = 3. The decrease of min contig length from 200 to 100 was to allow more sequences to be clustered by the TGI Clustering Tool (TGICL) (version 2.1) [84] and to be available for further analyses. Changes in reinforcement distance (from 75 to 85), min glue (from 2 to 3), and min ker cov (from 1 to 3) were made to increase the quality of resulting assemblies.

The assembled sequences (contigs) were clustered into unigenes using TGICL and the default settings, with the following changes: *I* (minimum overlap length) = 40, *c* (base quality cutoff of clipping) = 10, and *v* (maximum length of unmatched overhangs) = 20. TGICL-clustered contigs (>70% of sequence identity) were re-named as CLxxxx.Contigxx (x represents numeric numbers), as in CL9792.Contig11 and CL9792.Contig12. Non-clustered sequences (singleton) were labelled as Unigenexxx (as in Unigene107).

### 3.5. Unigenes Annotation and Coding DNA Sequence (CDS) Prediction

The assembled unigenes were annotated against the NR (release 2013_04) and NT (released 2013_04) databases, the Swiss-Prot database, the KEGG (release 2013_03) database, and the COG (release 2009_03) database using NCBI BLAST 2.2.28+ with an *E*-value < 10^−5^ (version 2.2.26). The software BLAST2GO (version 2.5.0) was used to retrieve GO annotations for unigenes annotated with the NR database. Subsequently, all unigenes annotated by GO were subjected to the software WEGO for functional classification. The software Path_finder (an internal version of BGI) was used to perform KEGG pathway annotation against the KEGG database. Unigenes failing to hit any in these databases were then subjected to the software ESTScan (version 3.0.2) for CDS prediction.

### 3.6. Detection of SSR and SNP Sites

MicroSatellite (MISA, http://pgrc.ipk-gatersleben.de/misa/) was used to identify SSR motifs in assembled unigenes with the following standards: mono repeats—12 units, dimer repeats—6 units, trimer repeats—5 units, tetramer repeats—5 units, pentamer repeats—5 units, and hexamer repeats—4 units. The software Primer3 (http://primer3.sourceforge.net/releases.php) was used to design primers for amplifying identified SSR sites in PCR products. To identify putative SNP sites, unigenes from all three caladium cultivars were combined to create a set of reference sequences. The SOAPsnp software (version 1.03) was employed to align unigenes of each cultivar against the combined set of reference sequences to call SNPs.

## Figures and Tables

**Table 1:** Summary of the HiSeq sequencing data of three caladium root transcriptomes.

Cultivars	Raw Reads	Clean Reads	Total Clean Nucleotides (nt)	Q20 Percentage (%)	N Percentage (%)	GC Percentage (%)
“Candidum”	68,000,078	61,439,682	6,143,968,200	96.84	0.01	49.20
“Gingerland”	50,980,474	46,148,492	4,614,849,200	96.97	0.01	49.47
“Miss Muffet”	67,583,936	61,034,284	6,103,428,400	96.92	0.01	49.84

**Table 2:** Summary of de novo assembly of caladium root transcriptomes.

	Sample	Total Number	Total Length (nt)	Mean Length (nt)	N50 Length (nt)	Total Consensus Sequences	Distinct Clusters	Distinct Singletons
Contig	“Candidum”	255,215	74,985,719	294	471	-	-	-
	“Gingerland”	232,333	69,427,118	299	492	-	-	-
	“Miss Muffet”	258,116	75,467,248	292	463	-	-	-
Unigene	“Candidum”	133,737	97,471,052	729	1525	133,737	46,962	86,775
	“Gingerland”	122,994	90,548,700	736	1514	122,994	43,721	79,273
	“Miss Muffet”	135,589	97,184,027	717	1487	135,589	47,844	87,745
	All	137,354	137,253,956	999	1755	137,354	62,353	75,001

**Table 3:** Summary of caladium unigenes annotated in different databases.

Databases	Number of Caladium Unigenes with Hits in Databases	Percentage of All Unigenes (%)
NR	68,827	50.11
NT	52,233	38.03
Swiss-Prot	47,907	34.88
KEGG	46,406	33.79
COG	31,417	22.87
GO	48,417	35.25
All	71,825	52.29

**Table 4:** The top 10 Kyoto Encyclopedia of Genes and Genomes (KEGG) pathways for the assembled caladium unigenes.

Rank	Pathway	Count (46,406)	Pathway ID	Function
1	Metabolic pathways	12,474	ko01100	Metabolism
2	RNA transport	5390	ko03013	Genetic Information Processing
3	Biosynthesis of secondary metabolites	4780	ko01110	Metabolism
4	mRNA surveillance pathway	4198	ko03015	Genetic Information Processing
5	Glycerophospholipid metabolism	3613	ko00564	Metabolism
6	Endocytosis	3563	ko04144	Cellular Processes
7	Ether lipid metabolism	3169	ko00565	Metabolism
8	Plant-pathogen interaction	2415	ko04626	Organismal Systems
9	Spliceosome	2024	ko03040	Genetic Information Processing
10	RNA degradation	1823	ko03018	Genetic Information Processing

**Table 5:** Summary of 98 putative disease resistance-related unigenes observed only in *Pythium*-resistant “Candidum”.

Functional Pathway	Putative Annotation	Number of Unigenes	Root-Specific
Signal perception	l-type lection receptor kinase (LecRK)	1	No
	Cysteine-rich receptor-like kinase (CRK)	1	No
	Receptor-like serine/threonine-protein kinases (STKs)	12	No
	Proline-rich receptor-like protein kinases (PERKs)	31	No
	Receptor-like protein kinases (RLKs)	5	No
Signal transduction	Calcium-dependent protein kinases (CPKs)	8	No
	Calmodulins (CaM) or calmodulins-like (CaM-like)	4	No
	Superoxide dismutase	5	No
	Mitogen-activated protein kinases (MAPKs)	8	No
Hormone regulation	Ethylene receptors	1	No
	*S*-adenosylmethionine (*S*-AdoMet)	1	No
	1-aminocyclopropane-1-carboxylic acid (ACC)	1	No
	Acyl-CoA oxidase (ACX1A)	1	No
Antimicrobial compounds	Phenylpropanoid biosythetic process	2	No
	Flavonoid	1	No
	Flavonoid 3	1	No
	Flavonoid 6-hydroxylase	1	No
	Flavonoid 3-monooxygenase	1	No
	Terpene synthase	4	No
	Tryptophan biosynthetic process	1	No
	Tryptophan 5-monooxygenase	5	No
	Tryptophan catabolic process	1	Yes
	Tryptophan 5-monooxygenase	1	No
	Tryptophan biosynthetic process	1	No

**Table 6:** Summary of simple sequence repeats (SSRs) identified in caladium root transcriptome sequences.

Number of Repeats	Mono-Nucleotide Repeats	Di-Nucleotide Repeats	Tri-Nucleotide Repeats	Quad-Nucleotide Repeat	Penta-Nucleotide Repeats	Hexa-Nucleotide Repeats
4	0	0	0	0	293	362
5	0	0	5183	356	53	4
6	0	3489	2857	87	0	1
7	0	2659	1431	0	0	0
8	0	2879	102	0	0	0
9	0	3501	7	0	0	0
10	0	2397	1	0	0	0
11	0	553	2	0	0	0
12	1086	18	0	0	0	0
13	543	4	0	0	0	0
14	336	4	0	0	0	0
15	178	0	0	0	0	0
16	109	0	0	0	0	0
17	66	0	0	0	0	0
18	52	0	0	0	0	0
19	37	0	0	0	0	0
20	41	0	0	0	0	0
21	54	0	0	0	0	0
22	38	0	0	0	0	0
23	53	0	0	0	0	0
24	1	0	0	0	0	0
SubTotal	2594	15,504	9583	443	346	367

**Table 7:** Summary of single nucleotide polymorphism (SNP) types detected in caladium root transcriptomes.

SNP Type	“Candidum”	“Gingerland”	“Miss Muffet”
Transition	169,687	147,579	162,919
A (adenine) ↔ G (guanine)	85,666	74,538	82,157
C (cytosine) ↔ T (thymine)	84,021	73,041	80,762
Transversion	112,041	97,106	107,298
A (adenine) ↔ C (cytosine)	27,274	23,705	25,952
A (adenine) ↔ T (thymine)	23,246	19,717	21,984
C (cytosine) ↔ G (guanine)	34,446	30,372	33,440
G (guanine) ↔ T (thymine)	27,075	23,312	25,922
Total	281,728	244,685	270,217

## Supplementary Materials

Supplementary materials can be found at www.mdpi.com/1422-0067/18/4/712/s1.

## Abbreviations

CDS	Coding DNA sequence
COG	Clusters of Orthologous Groups
GO	Gene Ontology
KEGG	Kyoto Encyclopedia of Genes and Genomes
NCBI	National Center for Biotechnology Information
NR	Non-redundant protein database (NCBI)
NT	Nucleotide sequence database (NCBI)
PAMP	Pathogen-associated molecular pattern
PTI	PAMP-triggered immunity
SNP	Single nucleotide polymorphism
SSR	Simple sequence repeat
USDA	United States Department of Agriculture

## References

[1] Cao Z., Mclaughlin M., Deng Z. (2014). Interspecific genome size and chromosome number variation shed new light on species classification and evolution in caladium. J. Am. Soc. Hortic. Sci..

[2] Gong L., Deng Z. (2011). Development and characterization of microsatellite markers for caladiums (*Caladium* Vent.). Plant Breed..

[3] Deng Z., Goktepe F., Harbaugh B.K., Hu J.G. (2007). Assessment of genetic diversity and relationships among caladium cultivars and species using molecular markers. J. Am. Soc. Hortic. Sci..

[4] Deng Z., Harbaugh B.K., Kelly R.O., Seijo T., McGovern R.J. (2005). Pythium root rot resistance in caladium cultivars. HortScience.

[5] Deng Z., Harbaugh B.K., Kelly R.O., Seijo T., McGovern R.J. (2005). Screening for resistance to pythium root rot among twenty-three caladium cultivars. HortTechnology.

[6] Ridings W.H., Hartman R.D. (1976). Pathogenicity of *Pythium myriotylum* and other species of *Pythium* to caladium derived from shoot-tip culture. Phytopathology.

[7] Bruehl G.W. (1983). Nonspecific genetic resistance to soil-borne fungi. Phytopathology.

[8] Goktepe F., Seijo T., Deng Z., Harbaugh B.K., Peres N.A. (2007). Toward breeding for resistance to fusarium tuber rot in caladium: Inoculation technique and sources of resistance. HortScience.

[9] Deng Z., Harbaugh B.K., Kelly R., Seijo T., McGovern R.J. (2004). Evaluation of caladium cultivars for resistance to pythium root rot. HortScience.

[10] Fawke S., Doumane M., Schornack S. (2015). Oomycete interactions with plants: Infection strategies and resistance principles. Microbiol. Mol. Biol. Rev..

[11] Sela-Buurlage M.B., Budai-Hadrian O., Pan Q., Carmel-Goren L., Vunsch R., Zamir D., Fluhr R. (2001). Genome-wide dissection of *Fusarium* resistance in tomato reveals multiple complex loci. Mol. Genet. Genom..

[12] Komatsu S., Yang G., Hayashi N., Kaku H., Umemura K., Iwasaki Y. (2004). Alterations by a defect in a rice G protein α subunit in probenazole and pathogen-induced responses. Plant Cell Environ..

[13] Wang X.L., Jiang N., Liu J.L., Liu W.D., Wang G.L. (2014). The role of effectors and host immunity in plant-necrotrophic fungal interactions. Virulence.

[14] Diener A.C., Ausubel F.M. (2005). Resistance to *Fusarium oxysporum* 1, a dominant *Arabidopsis* disease-resistance gene, is not race specific. Genetics.

[15] De Vleesschauwer D., Xu J., Hofte M. (2014). Making sense of hormone-mediated defense networking: From rice to *Arabidopsis*. Front. Plant Sci..

[16] Chan Y.L., Prasad V., Sanjaya, Chen K.H., Liu P.C., Chan M.T., Chiu-Ping C. (2005). Transgenic tomato plants expressing an *Arabidopsis* thionin (*thi2.1*) driven by fruit-inactive promoter battle against phytopathogenic attack. Planta.

[17] Ward J.A., Ponnala L., Weber C.A. (2012). Strategies for transcriptome analysis in non-model plants. Am. J. Bot..

[18] Xiao M., Zhang Y., Chen X., Lee E.J., Barber C.J.S., Chakrabarty R., Desgagne-Penix I., Haslam T.M., Kim Y.B., Liu E.W. (2013). Transcriptome analysis based on next-generation sequencing of non-model plants producing specialized metabolites of biotechnological interest. J. Biotechnol..

[19] Zhang H.B., Xia E.H., Huang H., Jiang J.J., Liu B.Y., Gao L.Z. (2015). De novo transcriptome assembly of the wild relative of tea tree (*Camellia taliensis*) and comparative analysis with tea transcriptome identified putative genes associated with tea quality and stress response. BMC Genom..

[20] He B., Gu Y.H., Tao X., Cheng X.J., Wei C.H., Fu J., Cheng Z.Q., Zhang Y.Z. (2015). De novo transcriptome sequencing of *Oryza officinalis* Wall ex Watt to identify disease-resistance genes. Int. J. Mol. Sci..

[21] Fu Y.Q., Esselink G.D., Visser R.G.F., van Tuyl J.M., Arens P. (2016). Transcriptome analysis of *Gerbera hybrida* including in silico confirmation of defense genes found. Front. Plant Sci..

[22] Kim J.E., Oh S.K., Lee J.H., Lee B.M., Jo S.H. (2014). Genome-wide SNP calling using next generation sequencing data in tomato. Mol. Cells.

[23] Guo Y.F., Wiegert-Rininger K.E., Vallejo V.A., Barry C.S., Warner R.M. (2015). Transcriptome-enabled marker discovery and mapping of plastochron-related genes in *Petunia* spp.. BMC Genom..

[24] Zheng X.F., Pan C., Diao Y., You Y.N., Yang C.Z., Hu Z.L. (2013). Development of microsatellite markers by transcriptome sequencing in two species of *Amorphophallus* (Araceae). BMC Genom..

[25] Li Z., Wang J., Zhang X., Xu L. (2015). Comparative transcriptome analysis of Anthurium “Albama” and its anthocyanin-loss mutant. PLoS ONE.

[26] Schliesky S., Gowik U., Weber A.P., Brautigam A. (2012). RNA-Seq assembly—Are we there yet?. Front. Plant Sci..

[27] Rajkumar H., Ramagoni R.K., Anchoju V.C., Vankudavath R.N., Syed A.U. (2015). De novo transcriptome analysis of *Allium cepa* L. (onion) bulb to identify allergens and epitopes. PLoS ONE.

[28] Cherukupalli N., Divate M., Mittapelli S.R., Khareedu V.R., Vudem D.R. (2016). De novo assembly of leaf transcriptome in the medicinal plant *Andrographis paniculata*. Front. Plant Sci..

[29] Ma X., Wang P., Zhou S., Sun Y., Liu N., Li X., Hou Y. (2015). De novo transcriptome sequencing and comprehensive analysis of the drought-responsive genes in the desert plant *Cynanchum komarovii*. BMC Genom..

[30] Wang Y., Li X., Zhou W., Li T., Tian C. (2016). De novo assembly and transcriptome characterization of spruce dwarf mistletoe *Arceuthobium sichuanense* uncovers gene expression profiling associated with plant development. BMC Genom..

[31] Zhukov V.A., Zhernakov A.I., Kulaeva O.A., Ershov N.I., Borisov A.Y., Tikhonovich I.A. (2015). De novo assembly of the pea (*Pisum sativum* L.) nodule transcriptome. Int. J. Genom..

[32] Xu L., Wang J., Lei M., Li L., Fu Y., Wang Z., Ao M., Li Z. (2016). Transcriptome analysis of storage roots and fibrous roots of the traditional medicinal herb *Callerya speciosa* (Champ.) schot. PLoS ONE.

[33] Tian S., Gu C., Liu L., Zhu X., Zhao Y., Huang S. (2015). Transcriptome profiling of *Louisiana iris* root and identification of genes involved in lead-stress response. Int. J. Mol. Sci..

[34] Dodds P.N., Rathjen J.P. (2010). Plant immunity: Towards an integrated view of plant-pathogen interactions. Nat. Rev. Genet..

[35] Boller T., Felix G. (2009). A renaissance of elicitors: Perception of microbe-associated molecular patterns and danger signals by pattern-recognition receptors. Annu. Rev. Plant Biol..

[36] Morris E.R., Walker J.C. (2003). Receptor-like protein kinases: The keys to response. Curr. Opin. Plant Biol..

[37] Mengiste T. (2012). Plant immunity to necrotrophs. Annu. Rev. Phytopathol..

[38] Shiu S.H., Karlowski W.M., Pan R.S., Tzeng Y.H., Mayer K.F.X., Li W.H. (2004). Comparative analysis of the receptor-like kinase family in *Arabidopsis* and rice. Plant Cell.

[39] Gish L.A., Clark S.E. (2011). The RLK/Pelle family of kinases. Plant J..

[40] Bouwmeester K., Govers F. (2009). *Arabidopsis*
l-type lectin receptor kinases: Phylogeny, classification, and expression profiles. J. Exp. Bot..

[41] Bouwmeester K., de Sain M., Weide R., Gouget A., Klamer S., Canut H., Govers F. (2011). The lectin receptor kinase LecRK-I.9 is a novel *Phytophthora* resistance component and a potential host target for a RXLR effector. PLoS Pathog..

[42] Wang Y., Cordewener J.H.G., America A.H.P., Shan W.X., Bouwmeester K., Govers F. (2015). *Arabidopsis* lectin receptor kinases LecRK-IX.1 and LecRK-IX.2 are functional analogs in regulating *Phytophthora* resistance and plant cell death. Mol. Plant Microbe Int..

[43] Chen K.G., Du L.Q., Chen Z.X. (2003). Sensitization of defense responses and activation of programmed cell death by a pathogen-induced receptor-like protein kinase in *Arabidopsis*. Plant Mol. Biol..

[44] Bourdais G., Burdiak P., Gauthier A., Nitsch L., Salojarvi J., Rayapuram C., Idanheimo N., Hunter K., Kimura S., Merilo E. (2015). Large-scale phenomics identifies primary and fine-tuning roles for CRKs in responses related to oxidative stress. PLoS Genet..

[45] Yang K., Rong W., Qi L., Li J., Wei X., Zhang Z. (2013). Isolation and characterization of a novel wheat cysteine-rich receptor-like kinase gene induced by *Rhizoctonia cerealis*. Sci. Rep. UK.

[46] Cao A.H., Xing L.P., Wang X.Y., Yang X.M., Wang W., Sun Y.L., Qian C., Ni J.L., Chen Y.P., Liu D.J. (2011). Serine/threonine kinase gene *Stpk-V*, a key member of powdery mildew resistance gene *Pm21*, confers powdery mildew resistance in wheat. Proc. Natl. Acad. Sci. USA.

[47] Sessa G., Martin G.B. (2000). Signal recognition and transduction mediated by the tomato Pto kinase: A paradigm of innate immunity in plants. Microbes Infect..

[48] Faris J.D., Zhang Z.C., Lu H.J., Lu S.W., Reddy L., Cloutier S., Fellers J.P., Meinhardt S.W., Rasmussen J.B., Xu S.S. (2010). A unique wheat disease resistance-like gene governs effector-triggered susceptibility to necrotrophic pathogens. Proc. Natl. Acad. Sci. USA.

[49] Haffani Y.Z., Silva-Gagliardi N.F., Sewter S.K., Grace Aldea M., Zhao Z., Nakhamchik A., Cameron R.K., Goring D.R. (2006). Altered expression of PERK receptor kinases in *Arabidopsis* leads to changes in growth and floral organ formation. Plant Signal. Behav..

[50] Silva N.F., Goring D.R. (2002). The proline-rich, extensin-like receptor kinase-1 (*PERK1*) gene is rapidly induced by wounding. Plant Mol. Biol..

[51] Malinovsky F.G., Fangel J.U., Willats W.G.T. (2014). The role of the cell wall in plant immunity. Front. Plant Sci..

[52] Lecourieux D., Raneva R., Pugin A. (2006). Calcium in plant defence-signalling pathways. New Phytol..

[53] Kobayashi M., Yoshioka M., Asai S., Nomura H., Kuchimura K., Mori H., Doke N., Yoshioka H. (2012). *StCDPK5* confers resistance to late blight pathogen but increases susceptibility to early blight pathogen in potato via reactive oxygen species burst. New Phytol..

[54] Takabatake R., Karita E., Seo S., Mitsuhara I., Kuchitsu K., Ohashi Y. (2007). Pathogen-induced calmodulin isoforms in basal resistance against bacterial and fungal pathogens in tobacco. Plant Cell Physiol..

[55] Levine A., Tenhaken R., Dixon R., Lamb C. (1994). H_2_O_2_ from the oxidative burst orchestrates the plant hypersensitive disease resistance response. Cell.

[56] Lehmann S., Serrano M., L’Haridon F., Tjamos S.E., Metraux J.P. (2015). Reactive oxygen species and plant resistance to fungal pathogens. Phytochemistry.

[57] Gupta S., Bhar A., Chatterjee M., Das S. (2013). *Fusarium oxysporum* f.sp *ciceri* race 1 induced redox state alterations are coupled to downstream defense signaling in root tissues of chickpea (*Cicer arietinum* L.). PLoS ONE.

[58] Kumar V., Parkhi V., Kenerley C.M., Rathore K.S. (2009). Defense-related gene expression and enzyme activities in transgenic cotton plants expressing an endochitinase gene from *Trichoderma virens* in response to interaction with *Rhizoctonia solani*. Planta.

[59] Ichimura K., Shinozaki K., Tena G., Sheen J., Henry Y., Champion A., Kreis M., Zhang S.Q., Hirt H., Wilson C. (2002). Mitogen-activated protein kinase cascades in plants: A new nomenclature. Trends Plant Sci..

[60] Chisholm S.T., Coaker G., Day B., Staskawicz B.J. (2006). Host-microbe interactions: Shaping the evolution of the plant immune response. Cell.

[61] Kong Q., Qu N., Gao M., Zhang Z., Ding X., Yang F., Li Y., Dong O.X., Chen S., Li X. (2012). The MEKK1-MKK1/MKK2-MPK4 kinase cascade negatively regulates immunity mediated by a mitogen-activated protein kinase kinase kinase in *Arabidopsis*. Plant Cell.

[62] Wang K.L.C., Li H., Ecker J.R. (2002). Ethylene biosynthesis and signaling networks. Plant Cell.

[63] Cho Y.H., Yoo S.D. (2015). Novel connections and gaps in ethylene signaling from the ER membrane to the nucleus. Front. Plant Sci..

[64] Thomma B.P.H.J., Eggermont K., Tierens K.F.M.J., Broekaert W.F. (1999). Requirement of functional ethylene-insensitiv*e* 2 gene for efficient resistance of *Arabidopsis* to infection by *Botrytis cinerea*. Plant Physiol..

[65] Hoffman T., Schmidt J.S., Zheng X.Y., Bent A.F. (1999). Isolation of ethylene-insensitive soybean mutants that are altered in pathogen susceptibility and gene-for-gene disease resistance. Plant Physiol..

[66] AbuQamar S., Chai M.F., Luo H.L., Song F.M., Mengiste T. (2008). Tomato protein kinase 1b mediates signaling of plant responses to necrotrophic fungi and insect herbivory. Plant Cell.

[67] Vijayan P., Shockey J., Levesque C.A., Cook R.J., Browse J. (1998). A role for jasmonate in pathogen defense of *Arabidopsis*. Proc. Natl. Acad. Sci. USA.

[68] Cohen Y., Gisi U., Niderman T. (1993). Local and systemic protection against *Phytophthora infestans* induced in potato and tomato plants by jasmonic acid and jasmonic methyl-ester. Phytopathology.

[69] Baetz U., Martinoia E. (2014). Root exudates: The hidden part of plant defense. Trends Plant Sci..

[70] Harborne J.B., Williams C.A. (2000). Advances in flavonoid research since 1992. Phytochemistry.

[71] Lanoue A., Burlat V., Henkes G.J., Koch I., Schurr U., Rose U.S.R. (2010). De novo biosynthesis of defense root exudates in response to *Fusarium* attack in barley. New Phytol..

[72] Arfaoui A., El Hadrami A., Mabrouk Y., Sifi B., Boudabous A., El Hadrami I., Daayf F., Cherif M. (2007). Treatment of chickpea with *Rhizobium* isolates enhances the expression of phenylpropanoid defense-related genes in response to infection by *Fusarium oxysporum* F. sp. *ciceris*. Plant Physiol. Biochem..

[73] Balmer D., de Papajewski D.V., Planchamp C., Glauser G., Mauch-Mani B. (2013). Induced resistance in maize is based on organ-specific defence responses. Plant J..

[74] Vaughan M.M., Wang Q., Webster F.X., Kiemle D., Hong Y.J., Tantillo D.J., Coates R.M., Wray A.T., Askew W., O’Donnell C. (2013). Formation of the unusual semivolatile diterpene rhizathalene by the *Arabidopsis* class I terpene synthase TPS08 in the root stele is involved in defense against belowground herbivory. Plant Cell.

[75] Steeghs M., Bais H.P., de Gouw J., Goldan P., Kuster W., Northway M., Fall R., Vivanco J.M. (2004). Proton-transfer-reaction mass spectrometry as a new tool for real time analysis of root-secreted volatile organic compounds in *Arabidopsis*. Plant Physiol..

[76] Kapulnik Y., Delaux P.M., Resnick N., Mayzlish-Gati E., Wininger S., Bhattacharya C., Sejalon-Delmas N., Combier J.P., Becard G., Belausov E. (2011). Strigolactones affect lateral root formation and root-hair elongation in *Arabidopsis*. Planta.

[77] Stotz H.U., Sawada Y., Shimada Y., Hirai M.Y., Sasaki E., Krischke M., Brown P.D., Saito K., Kamiya Y. (2011). Role of camalexin, indole glucosinolates, and side chain modification of glucosinolate-derived isothiocyanates in defense of *Arabidopsis* against *Sclerotinia sclerotiorum*. Plant J..

[78] Millet Y.A., Danna C.H., Clay N.K., Songnuan W., Simon M.D., Werck-Reichhart D., Ausubel F.M. (2010). Innate immune responses activated in *Arabidopsis* roots by microbe-associated molecular patterns. Plant Cell.

[79] Ching A., Caldwell K.S., Jung M., Dolan M., Smith O.S., Tingey S., Morgante M., Rafalski A.J. (2002). SNP frequency, haplotype structure and linkage disequilibrium in elite maize inbred lines. BMC Genet..

[80] Agarwal M., Shrivastava N., Padh H. (2008). Advances in molecular marker techniques and their applications in plant sciences. Plant Cell Rep..

[81] Vleeshouwers V., Oliver R. (2014). Effectors as tools in disease resistance breeding against biotrophic, hemibiotrophic, and necrotrophic plant pathogens. Mol. Plant Microbe Interact..

[82] Ashkani S., Rafii M.Y., Shabanimofrad M., Miah G., Sahebi M., Azizi P., Tanweer F.A., Akhtar M.S., Nasehi A. (2015). Molecular breeding strategy and challenges towards improvement of blast disease resistance in rice crop. Front. Plant Sci..

[83] Grabherr M.G., Haas B.J., Yassour M., Levin J.Z., Thompson D.A., Amit I., Adiconis X., Fan L., Raychowdhury R., Zeng Q. (2011). Full-length transcriptome assembly from RNA-Seq data without a reference genome. Nat. Biotechnol..

[84] Pertea G., Huang X., Liang F., Antonescu V., Sultana R., Karamycheva S., Lee Y., White J., Cheung F., Parvizi B. (2003). TIGR gene indices clustering tools (TGICL): A software system for fast clustering of large est datasets. Bioinformatics.

